# Integrated analysis highlights the significance role of ITGAL in lung adenocarcinoma

**DOI:** 10.1111/jcmm.18289

**Published:** 2024-04-13

**Authors:** Zengtuan Xiao, Zhe Nian, Mengzhe Zhang, Zuo Liu, Zhe Liu, Zhenfa Zhang

**Affiliations:** ^1^ Department of Immunology, School of Basic Medical Sciences, Department of Lung Cancer Surgery, Tianjin Lung Cancer Center Tianjin Medical University Tianjin China

**Keywords:** immunotherapy, ITGAL, lung adenocarcinoma, NK cell, prognosis

## Abstract

Integrin alpha L (ITGAL), a member of the integrin family, is associated with carcinogenesis and immune regulation. However, the biological functions of ITGAL in lung adenocarcinoma (LUAD) remain poorly understood. In this study, we utilized the TCGA dataset to analyse ITGAL mRNA expression in LUAD and examined its correlation with clinical prognosis. Three‐dimensional (3D) Matrigel culture, 5‐bromodeoxyuridine (BrdU) ELISA, wound‐healing migration and cell adherence assays were used to demonstrate the potential role of ITGAL in LUAD progression. Additionally, we analysed single‐cell sequencing data of LUAD to determine the expression and biological function of ITGAL. Our research revealed that the expression of ITGAL in LUAD samples is an independent predictor of prognosis. Patients with high expression of ITGAL had significantly better overall survival (OS), progression‐free survival (PFS) and disease‐specific survival (DSS) compared to the low‐expression group. Meanwhile, the expression of ITGAL suppressed malignant progression in LUAD cells. Functional enrichment analyses showed that ITGAL was significantly correlated with cell immune response and immune checkpoint, consistent with the analysis of single‐cell sequencing in paired samples of normal and tumour. Furthermore, we confirmed that ITGAL expression affect the tumour microenvironment (TME) through regulation of the expression of cytokines in NK cells of LUAD. In summary, ITGAL is a prognostic biomarker for LUAD patients, and it repressed malignant progression in LUAD cells. Moreover, ITGAL expression also enhanced the effect of immunotherapy and may be an important target in LUAD therapy.

## 1 BACKGROUND

Lung cancer was the most common cause of cancer‐related fatalities worldwide in 2022.^1^ LUAD is the most common histological form and shows a great deal of heterogeneity.^2^ The 5‐year survival rate for patients with LUAD is very low, even with advances in targeted therapy, immunotherapy, radiation, chemotherapy and surgery.^3^ The field of LUAD treatment has changed recently with the advent of immunotherapies that target immunological checkpoints, producing impressive therapeutic effects.^4^, ^5^ Current clinical practice uses a number of biomarkers, including PD‐L1, CTLA‐4 and tumour mutation burden (TMB), to predict immunotherapy response.^6^, ^7^, ^8^, ^9^ Nonetheless, only a portion of LUAD patients benefit from immunotherapy, and these biomarkers are not entirely capable of capturing the diverse TME.^10^ Thus, finding novel biomarkers that accurately predict prognosis and responsiveness to therapy is critical.

The chromosome's 16p11.2 region contains the gene ITGAL, sometimes referred to as CD11a, which codes for an integrin that is a part of LFA‐1. This integrin has expression in the immune cells and controls lymphocyte co‐stimulation signalling and intercellular adhesion,^11^, ^12^ which is important for angiogenesis and the development of cancer.^13^, ^14^ Furthermore, ITGAL takes part in immunological responses and contributes to the pathogenesis of a number of malignancies, including gliomas, breast, colorectal, gastric and head–neck squamous cell carcinomas.^15^, ^16^, ^17^, ^18^, ^19^ These studies propose that ITGAL may considerably affect the cancer progression, leading to its potential use as a novel target in cancer therapy. Given the established involvement of ITGAL in neoplastic progression and the immune responses, we speculated that ITGAL may play an important role in LUAD and affect the microenvironment of LUAD.

**FIGURE 1:**
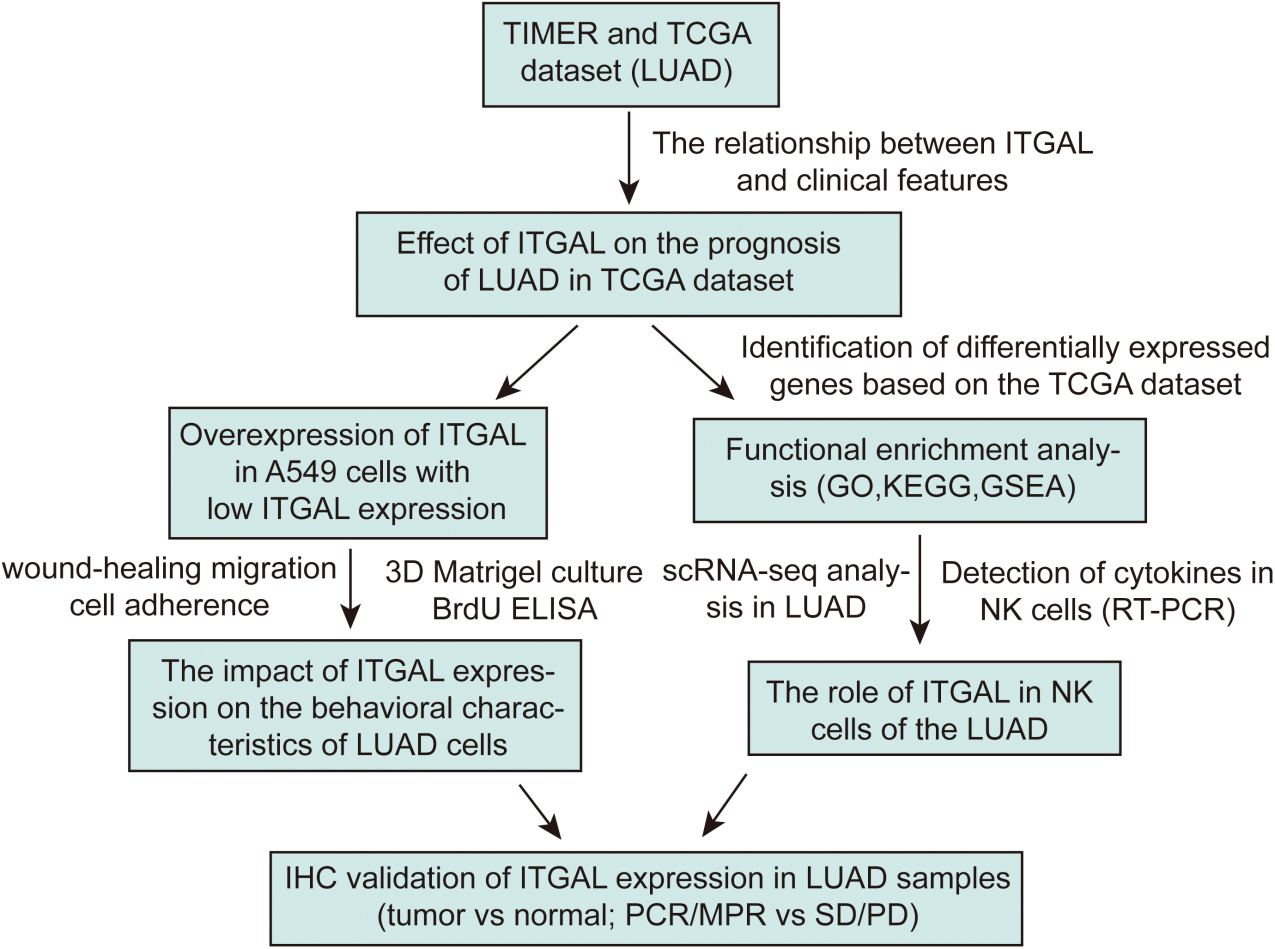
The flow diagram of the study.

In this study, we examined the expression of ITGAL and its link to the prognosis of LUAD patients, by utilizing both TCGA and single‐cell datasets.^20^ We found that. ITGAL may considerably repress the malignant progression of LUAD cells. Moreover, we found a relationship between ITGAL expression and the immune microenvironment of LUAD, and further illustrated that ITGAL can impact the cytokine levels in NK cells within LUAD via single‐cell RNA sequencing data. Finally, we verified the role of ITGAL by immunohistochemistry on tissue samples, suggesting that ITGAL can serve as a clinical marker. Figure 1 demonstrates the organization of the study's workflow. This study provides insights into the malignant progression and immunotherapy‐related roles of ITGAL in LUAD. It also provides solid experimental and theoretical evidence for developing effective therapy strategies in LUAD, and ITGAL might be a novel target for the treatment of LUAD, particularly immunotherapy.

## 2 METHODS

### 2.1 Data acquiring and cleaning

Transcriptome sequencing data and clinical information from LUAD patients were downloaded from the Cancer Genome Atlas (TCGA) LUAD cohort (https://portal.gdc.cancer.gov/). Data cleaning was conducted using Perl (v5.3.0) and R software (v4.2.3). The LUAD single‐cell dataset used in this study was collected from Bischoff et al.'s research.^20^ The final dataset consisted of 598 samples (539 tumour and 59 normal) for the TCGA bulk RNA‐seq analysis and 8 pairs of tumour and normal tissues for the single‐cell analysis.

### 2.2 Single‐cell processing

We visualized the processed data of scRNA‐seq using Seurat (v3.1) to cluster cell. Initially, the filtered matrix from the samples identified by Cell Ranger Count were inputted into Seurat. Following quality control and data harmonization, cell clustering was performed with Uniform Manifold Approximation and Projection (UMAP). Differentiated gene expression between two cell groups was identified using the Wilcoxon Rank Sum test via the FindMarkers function in Seurat software. Differentially expressed genes were identified against the average expression of all other clusters for each individual cluster. FindMarkers was used to calculate the log‐fold changes of all genes (‘thresh. test = 1.5’, min.pct = 0.25), which were then assessed by the GSEA analysis. The gseGO function of ClusterProfiler package (v3.18.1) was employed, using GO Biological Process ontology gene sets. For manual annotation of different clusters, the ‘singleR’ package was utilized with the default parameter.

### 2.3 The differential expression and clinical characteristics of ITGAL

The difference in ITGAL expression levels in pan‐cancer was acquired from TIMER (Tumour Immune Estimation Resource) (https://cistrome.shinyapps.io/timer/). The expression of ITGAL across various clinical features in the TCGA dataset was analysed and visualized using the ‘limma’, ‘ggplot2’ and ‘ggpubr’ R packages. Heatmap and correlation analyses examining the relationship between ITGAL expression and underlying clinical parameters were performed using the ‘limma’, ‘ggpubr’ and ‘ComplexHeatmap’ R packages. Survival curves were calculated using ‘survival’ and ‘survminer’ packages, and the median of ITGAL mRNA expression was used as a cut‐off value. A nomogram combining expression of ITGAL and clinic pathological risk factors was constructed by ‘rms’ package. Calibration curves were plotted to assess the calibration of the radiomics nomogram, as accompanied by the Hosmer–Lemeshow test. Cox proportional hazards models estimating the hazard ratio were established to determine whether ITGAL is associated with survival. Hazard ratios (HRs) with 95% confidence intervals and log‐rank *p*‐value were calculated via univariate survival analysis, and a *p* < 0.05 was considered statistically significant. Multivariate Cox regression analysis was performed on the variables that reached the significance level of *p* < 0.05 in univariate analysis.

### 2.4 Cells

A549 cells were obtained from ATCC within the past 10 years and maintained in ATCC recommended media supplemented with 10% FBS, 100 U of penicillin/mL and 100 μg of streptomycin/mL. All experiments were performed within 1 month after thawing early‐passage cells.

### 2.5 Immunoblots

Proteins were separated by10% Bis–Tris gels using 1 × SDS running buffer and transferred to PVDF membranes. Blots were blocked in TBS containing 5% milk and incubated overnight with ITGAL (sc‐374172, santa) and GAPDH (AC002, ABclonal) antibodies. The next day, blots were washed three times with TBS containing 1% Tween and exposed to the secondary antibody for 1 h at room temperature. Blots were washed three times followed by incubation in luminol‐based substrate for HRP‐catalysed detection. Luminescence was captured on an Amersham Imager 600.

### 2.6 Adhesion assay

The fibronectin (10 μg/mL) was added to a 6‐well plate and incubated at 4°C overnight. After the coating process, cultured cells were seeded onto the coated dishes for 30 min. The adhered cells were fixed using 4% paraformaldehyde, and then stained with 0.005% crystal violet, photographed and counted.

### 2.7 3D Matrigel culture

Detached cells were mixed into 5% Matrigel with prechilled 2% FBS‐RPMI 1640, and added to a 24‐well ultra‐low attachment plate (Corning) at 37°C for and 5% CO_2_ for 7–14 days. The culture was replenished every 2 days.

### 2.8 Brdu cell proliferation assays

For the cell proliferation assays, 1 × 10^3^ cells were cultured in 96‐well plates for 24 h. 5‐Bromo‐2′‐deoxyuridine was added according to the manufacturer's instructions of the kit (Cell Proliferation ELISA (11647229001, Roche)). The absorbance was measured at 450 nm on an enzyme‐labelled instrument.

### 2.9 Cell invasion assays

A 500 μL of culture medium was added to cell plate before transferring Transwell inserts with 8 μm membrane (BD Biosciences) coated with 20% Matrigel (Corning). A total of 2 × 10^5^ cells suspended in serum‐free medium were added to the upper chamber of the Transwell inserts. After incubation at 37°C for 24 h, the cells that had invaded through the membrane to the lower surface were fixed with 4% paraformaldehyde, and then stained with 0.005% crystal violet, photographed and counted.

### 2.10 Identification of DEGs and functional analysis of ITGAL‐related genes

All data were processed using R software. The ‘limma’ package was employed to identify differentially expressed genes (DEGs) between the ITGAL high‐expression and low‐expression groups in the TCGA dataset. DEGs were defined as having an adjusted *p* < 0.05 and an absolute log2 fold change (logFC) >1. Heatmaps of the DEGs were generated and displayed using the VennDiagram package. Gene ontology (GO) and Kyoto Encyclopedia of Genes and Genomes (KEGG) pathway enrichment analyses were executed on the DEGs correlated to ITGAL. The ‘clusterProfiler’ R package was employed for this purpose. Visualization of the relevant results was conducted using the ‘enrichplot’ package in R software.

### 2.11 Gene set enrichment analysis

To further examine the potential functional ramifications of ITGAL, we divided individuals with lung adenocarcinoma (LUAD) from the TCGA dataset into two groups based on the median expression level of ITGAL. We used GSEA (4.2.2) (www.gsea‐msigdb.org/gsea/index.jsp) to investigate whether genes differentially regulated between the two groups were enriched in cancer‐related biological pathways. We selected the annotated gene sets c2.cp.kegg.v2022.1.Hs.symbols.gmt and h.all.v2022.1.Hs.symbols.gmt as the reference gene set. FDR (*q*‐value) <0.05 and *p* < 0.05 were used to defined statistically significant enrichment. Finally, the log‐fold change (FC) values calculated and imported into R software for visualization analysis using the R packages ‘clusterProfiler’ and ‘enrichplot’.

### 2.12 Flow cytometry

NK92 cell staining was performed according to standard protocols. Antibodies against ITGAL (Abcam, ab52895) were used according to manufacturer recommendations. Cells were washed with PBS with 2% FBS, and were sorted on a BD FACS Aria II and data were analysed by FlowJo 10.6.1.

### 2.13 RT‐PCR

Total RNA was isolated with Trizol (Invitrogen) and reverse transcribed with oligo dT. One microgram of cDNA was used for PCR reactions. Primers used for GAPDH, IFI16, IFNG and TNF PCR are listed in the Table S1.

### 2.14 Correlation between ITGAL and tumour immune checkpoints

To clarify the underlying immunomodulatory mechanism of ITGAL, we evaluated the checkpoints in LUAD samples in TCGA dataset, using the CIBERSORT algorithm and then visualized using R.

### 2.15 TMB analysis

The somatic variants data of patients with lung adenocarcinoma (LUAD) were analysed and visualized using the maftools package, with the MAF file pipeline ‘muse’.^21^ Subsequently, we calculated the tumour mutation burden (TMB) for each patient by determining the number of mutations per million bases.

### 2.16 Predicting the patients' response to ICI

The Cancer Immunome Atlas (https://tcia.at/) conducted an analysis of the immune landscapes and antigenomes of 20 solid tumours. Tumour immunogenicity was quantitatively scored on a scale from 0 to 10, called the immunophenoscore (IPS).^22^ The IPS value exhibited a positive correlation with tumour immunogenicity and has been validated as a predictor of patient response to immune checkpoint inhibitor (ICI) treatment. We obtained IPS data for subsequent analysis. We also compared the mRNA levels of immune checkpoint molecules and their ligands between the high‐risk and low‐risk groups.

### 2.17 Immunohistochemistry

This study was conducted on 20 paired lung adenocarcinoma (LUAD) patients with normal and tumour specimens, 128 tumour specimens and 20 tumour specimens obtained after immunotherapy from Tianjin Medical University Cancer Institute and Hospital. Immunohistochemistry (IHC) was performed to investigate the expression of ITGAL using anti‐ITGAL antibody (Abcam, cat#ab78058). The staining intensity was classified as negative (0), weak (1), moderate (2), or strong (3), while the percentage of positive cells was divided into five levels: 0 indicating no positive cells, 1 indicating 1%–10% positive cells, 2 indicating 11%–50% positive cells, 3 indicating 51%–80% positive cells and 4 indicating 81%–100% positive cells. The final score was obtained by adding the staining intensity and the number of positive cells together.

### 2.18 Statistical analyses

Statistical analyses were performed using R (v4.2.3). The Kruskal–Wallis test was applied to analyse continuous variable data, while the Chi‐square test was used to examine the correlation between the expression levels of ITGAL and clinicopathological characteristics of the LUAD patients. The Cox regression model from the survival package was used to estimate the risk ratio (HR) and 95% confidence interval (CI). Survival analysis was performed using the Kaplan–Meier curve. A significance level of *p* < 0.05 (two‐tailed) was considered statistically significant for all analyses.

## 3 RESULTS

### 3.1 Expression of ITGAL in pan‐cancer analysis and its correlation with clinicopathological features of LUAD

**FIGURE 2:**
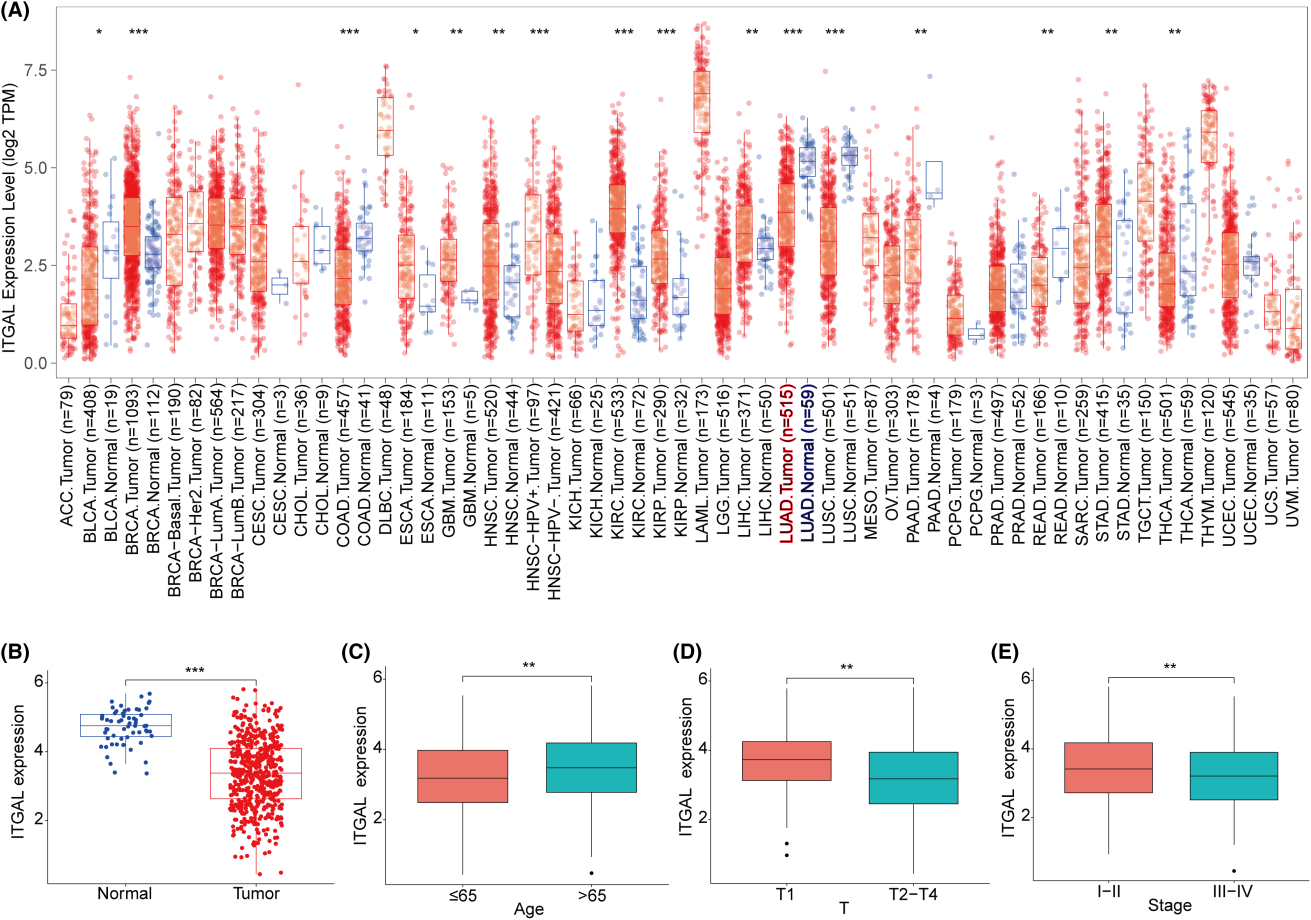
ITGAL expression in the LUAD. (A) ITGAL expression in different cancer types according to TCGA dataset was assessed using TIMER. (***p < 0.001, **p < 0.01, *p < 0.05. Blue and red represent normal and tumour samples respectively). (B) ITGAL mRNA level in non‐paired tumour samples in the LUAD based on TCGA dataset, ***p < 0.001. (C–E) Boxplots depict the expression of ITGAL in patients of the TCGA dataset, as categorized based on age (C), T stage (D) or stages (E). **p < 0.01.

We conducted a pan‐cancer analysis of ITGAL in tumours through the TIMER database, and the results showed that ITGAL was significantly more highly expressed in normal lung tissues than in the lung adenocarcinoma (LUAD) tissues (Figure 2A). TCGA dataset also corroborated that the expression of ITGAL was significantly higher in normal tissues than in LUAD tissues, and similar results were also obtained in paired LUAD tissues (Figure 2B; Figure S1A,B). Then, we analysed the relationship between the expression level of ITGAL and clinical features and found that ITGAL expression level was significantly associated with the age, gender, T stage and clinical stage (Figure 2C–E; Figure S1C). However, the expression level of ITGAL was not related to the N stage and M stage (Figure S1D–F).

### 3.2 Effect of ITGAL on the prognosis of LUAD

**FIGURE 3:**
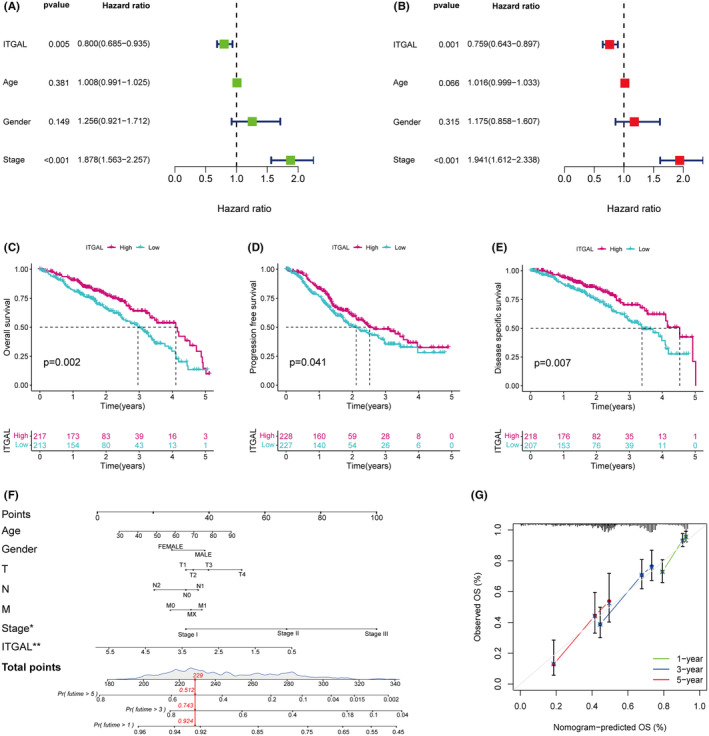
Prognosis analysis of ITGAL in the LUAD. (A) The Cox proportional regression analysis using the TCGA dataset, with the ITGAL included. (B) Multivariate Cox proportional regression analysis in the TCGA dataset, with the ITGAL included. (C–E) Kaplan–Meier plots of overall survival (C), progression free survival (D) and disease specific survival (E) in LUAD patients based on the expression levels of ITGAL. (F) Prognostic nomogram for LUAD. According to the seven variables (age, sex, stage, pathologic_T, pathologic_N, pathologic_M and ITGAL) in the model, 7 corresponding ‘points’ values can be obtained, and the ‘total points’ can be calculated by summing them. Therefore, the 1‐/3‐/5‐year survival rate of patients can be predicted. (G) Calibration curve of the nomogram at 1‐/3‐/5‐year OS. Nomogram‐predicted OS probability is presented on the *x*‐axis; actual survival is presented on the *y*‐axis. Good concordance for 1‐year, 3‐year and 5‐year survival of the nomogram‐predicted probability with the actual survival was obtained.

Next, we assess the prognostic significance of ITGAL. The median of ITGAL mRNA expression levels was used as a cut‐off value and divided patients in the TCGA dataset into ITGAL high expression and ITGAL low expression groups. Then, we conducted univariate and multivariate Cox regression analyses and found that ITGAL was an independent prognostic factor for overall survival in LUAD (Figure 3A,B). In addition, survival analysis showed that patients with low‐expression group had worse overall survival (OS) (*p* = 0.002), progression‐free survival (PFS) (*p* = 0.041) and disease‐specific survival (DSS) (*p* = 0.007) than patients in the high‐expression group (Figure 3C–E), while the expression of ITGAL did not affect disease‐free survival (DFS) (*p* = 0.555) (Figure S1G).

To establish a predictive tool for quantitative analysis of OS in LUAD patients, we development a nomogram model. The nomogram model included the following risk factors for OS: Age, Gender, T stage, N stage, M stage, TNM stage and ITGAL expression (Figure 3F). The Calibration plots showed that the observed line and the ideal line were closed in the calibration curve, demonstrating the nomogram was accurate and reliable (Figure 3G). These results indicate that ITGAL was a risk factor for LUAD patients and its low expression level correlates with extremely poor survival rates in LUAD patients.

### 3.3 The expression of ITGAL affected the malignant progression in LUAD cells

**FIGURE 4:**
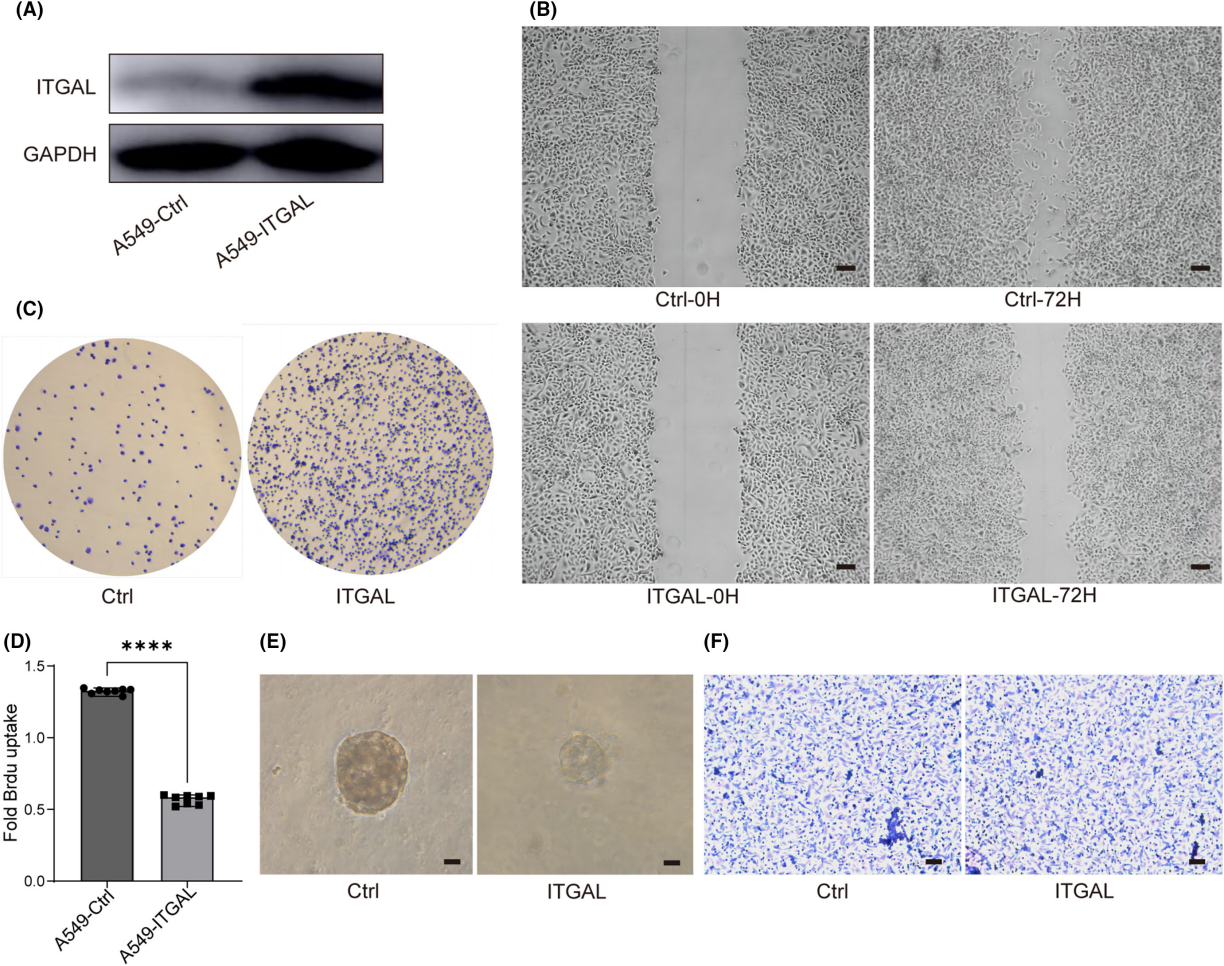
ITGAL overexpression affects the biological function of the LUAD cells. (A) Immunoblot analysis of ITGAL expression in A549 cells. (B) Images of wound‐healing assay showed the motility of control and ITGAL‐expressing A549 cells. Scale bars represent 20 μm. (C) ITGAL‐expressing A549 cells were plated on fibronectin‐coated plates. After 15 min, the attached cells were counted. (D) BrdU incorporation assay of control and ITGAL‐expressing A549 cells. ****p < 0.0001, unpaired two‐tailed Student's *t*‐test. (E) Phase‐contrast micrographs of control and ITGAL‐expressing A549 cells cultured on Matrigel for 8 days. Scale bars represent 20 μm. (F) Control and ITGAL‐expressing A549 cells were subjected to an invasion assay. Scale bars represent 20 μm.

Next, we overexpressed ITGAL in human lung adenocarcinoma A549 cells and explored the effect of ITGAL (Figure 4A). We found that ITGAL overexpression reduced motility of A549 in wound‐healing migration assays (Figure 4B). The cell detachment from substrate also increased after the expression of ITGAL (Figure 4C). These data indicated a function of ITGAL in suppressing cancer cell migratory and adhesive capability. Moreover, ITGAL overexpression showed significant inhibition effect on cell proliferation as evaluated by bromodeoxyuridine (BrdU) incorporation analysis (Figure 4D). In three‐dimensional (3D) Matrigel culture, ITGAL overexpression restrained the growth of clones, confirming the role of ITGAL in cell proliferation (Figure 4E). However, there was no significant change observed in the invasive ability of ITGAL‐expressing A549 cells though Matrigel (Figure 4F). These results collectively confirmed that ITGAL is a potential tumour suppressor in LUAD.

### 3.4 Functional enrichment analysis of DEGs from high‐expression ITGAL and low‐expression ITGAL groups

**FIGURE 5:**
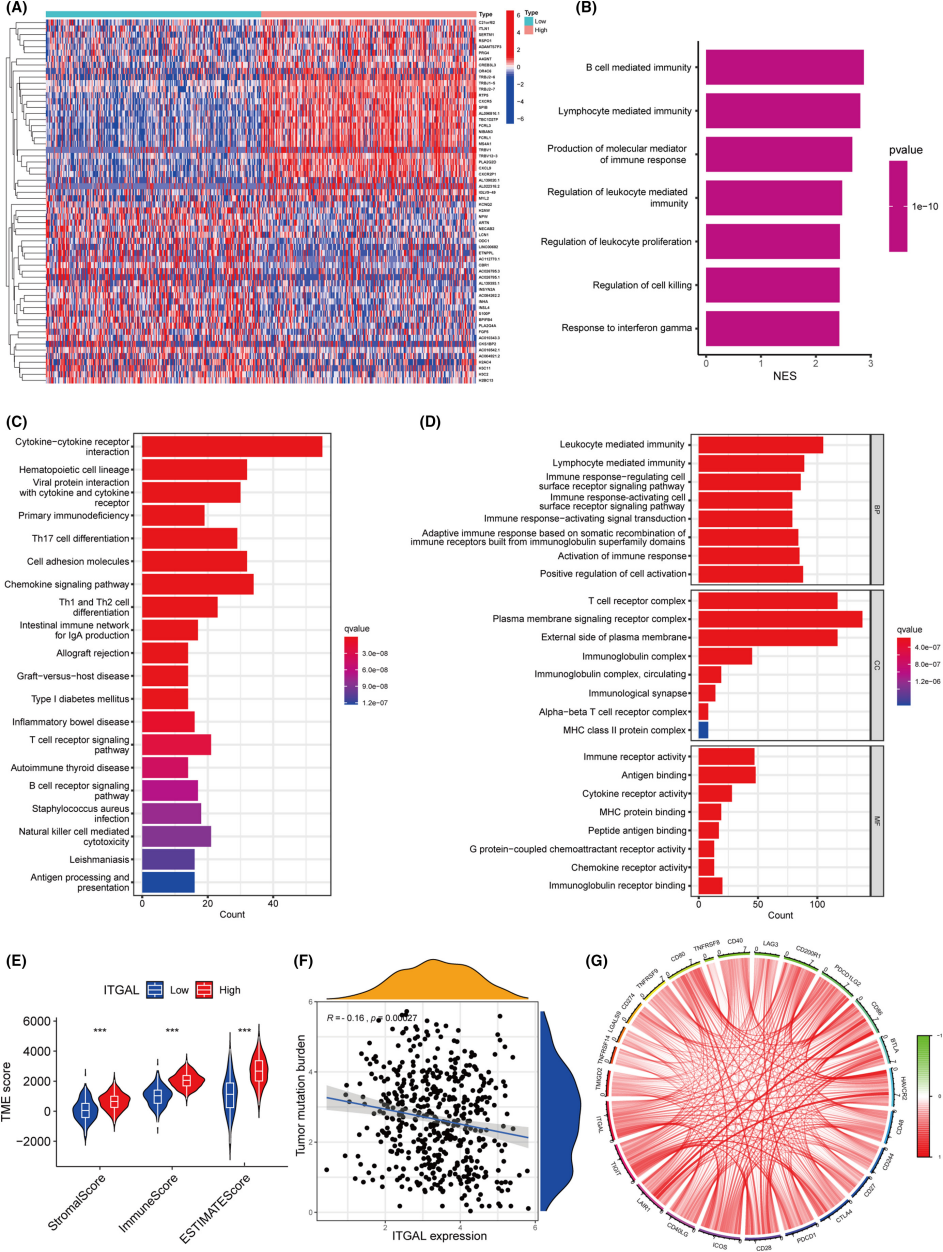
ITGAL expression associates with the TME in the LUAD. (A) Heatmap of differentially expressed genes (DEGs) between high‐ and low‐expression groups. (B–D) GSEA (B), KEGG (C) and GO (D) analysis revealed that ITGAL expression is involved in the immune response. (E) The correlation of ITGAL with the immune score, estimate score and stromal score. (F) The correlation of ITGAL with the tumour mutation burden. (G) Expression of ITGAL is related to a panel of immune checkpoint genes.

To further explore the function of ITGAL, we identified 1069 up‐regulated and 46 down‐regulated genes based on limma analysis (Figure 5A) in LUAD according to the TCGA dataset. GSEA, KEGG and GO analyses were conducted to identify the differential gene sets, which might be associated with the function of ITGAL in LUAD. GSEA showed that ITGAL expression involved in the immune response, including B cell mediated immunity, lymphocyte mediated immunity and regulation of cell killing (Figure 5B). The KEGG enrichment results included cytokine–cytokine receptor interaction, primary immunodeficiency and natural killer cell mediated cytotoxicity pathways (Figure 5C). Biological process (BP) included leukocyte mediated immunity, lymphocyte mediated immunity and activation of immune response; cellular components (CC) included T cell receptor complex, Alpha‐beta T cell receptor complex and MHC class II protein complex; molecular function (MF) included immune receptor activity, cytokine receptor activity and immunoglobulin receptor binding (Figure 5D). Taken together, these findings demonstrated that ITAGL is positively associated with the immune responses in LUAD.

### 3.5 The expression of the ITGAL has been linked to the TME in the LUAD

To further determine the function of ITGAL in immune responses of LUAD, we used ESTIMATE analysis. The expression of ITGAL was correlated significantly with stromal score, immune score and estimate score and patients with high ITGAL expression were found to score higher on TME (Figure 5E). In addition, the expression of ITGAL was correlated negatively with the tumour mutation burden (Figure 5F). Furthermore, the expression of ITGAL correlated positively with the expression of immune checkpoint genes such as PD‐1, PD‐L1 and CTLA4 (Figure 5G; Figure S1H,I). These results showed that ITGAL contributes to the reestablishment of the TME in LUAD.

### 3.6 Single cell RNA‐Seq analysis in human primary LUAD and their paired normal tissues

**FIGURE 6:**
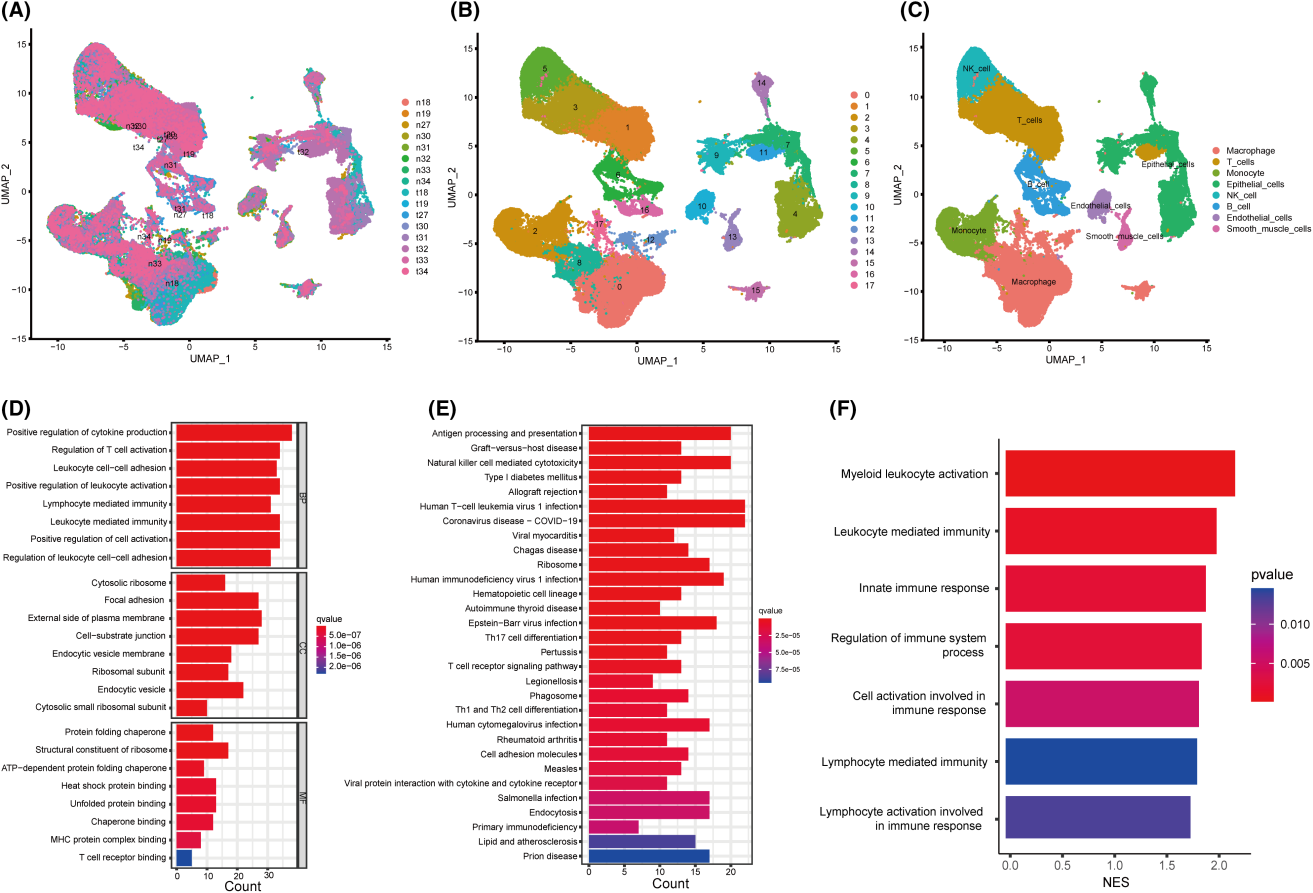
Single cell RNA‐seq reveals the role of ITGAL. (A) Uniform manifold approximation and projection (UMAP) plots for the 99,867 high‐quality cells showing sample origin. (B) UMAP plots of 99,867 high‐quality cells showing transcriptomic cluster. (C) UMAP plots of 99,867 high‐quality cells showing the cell type classification. (D–F) GO (D), KEGG (E) and GSEA (F) analysis revealed that ITGAL expression is involved in different pathways in the tumour samples.

Based on the scRNA‐seq data of Bischoff P et al.,^20^ we obtained gene expression profiles of 99,867 cells from 8 primary LUAD samples and matched normal tissues for further analysis (Figure 6A; Figure S2A,B). We grouped the cells according to expression profiles using the uniform manifold approximation and projection (UMAP) analysis, and identified 18 transcriptionally distinct cell clusters (Figure 6B). Subsequently, the cell identity of each cluster was annotated using a reference dataset from the Human Primary Cell Atlas (Figure 6C). To decipher the molecular features of ITGAL‐expressing cells that were distinct from those of the other cells in tumour samples, we obtained the different marker genes by using the FindAllMarkers. Then, the marker genes of ITGAL‐expressing cells were enriched for GO and KEGG functions. We found that the marker genes of ITGAL‐expressing cells are associated with positive regulation of cytokine production, lymphocyte mediated immunity and natural killer cell mediated cytotoxicity (Figure 6D,E), consistent with the results of the bulk RNA‐seq of TCGA dataset. In addition, gene set enrichment analysis (GSEA) revealed cell activation involved in the immune response in ITGAL‐expressing cells versus other cells in tumours (Figure 6F).

### 3.7 The role of ITGAL in NK cells of the LUAD

**FIGURE 7:**
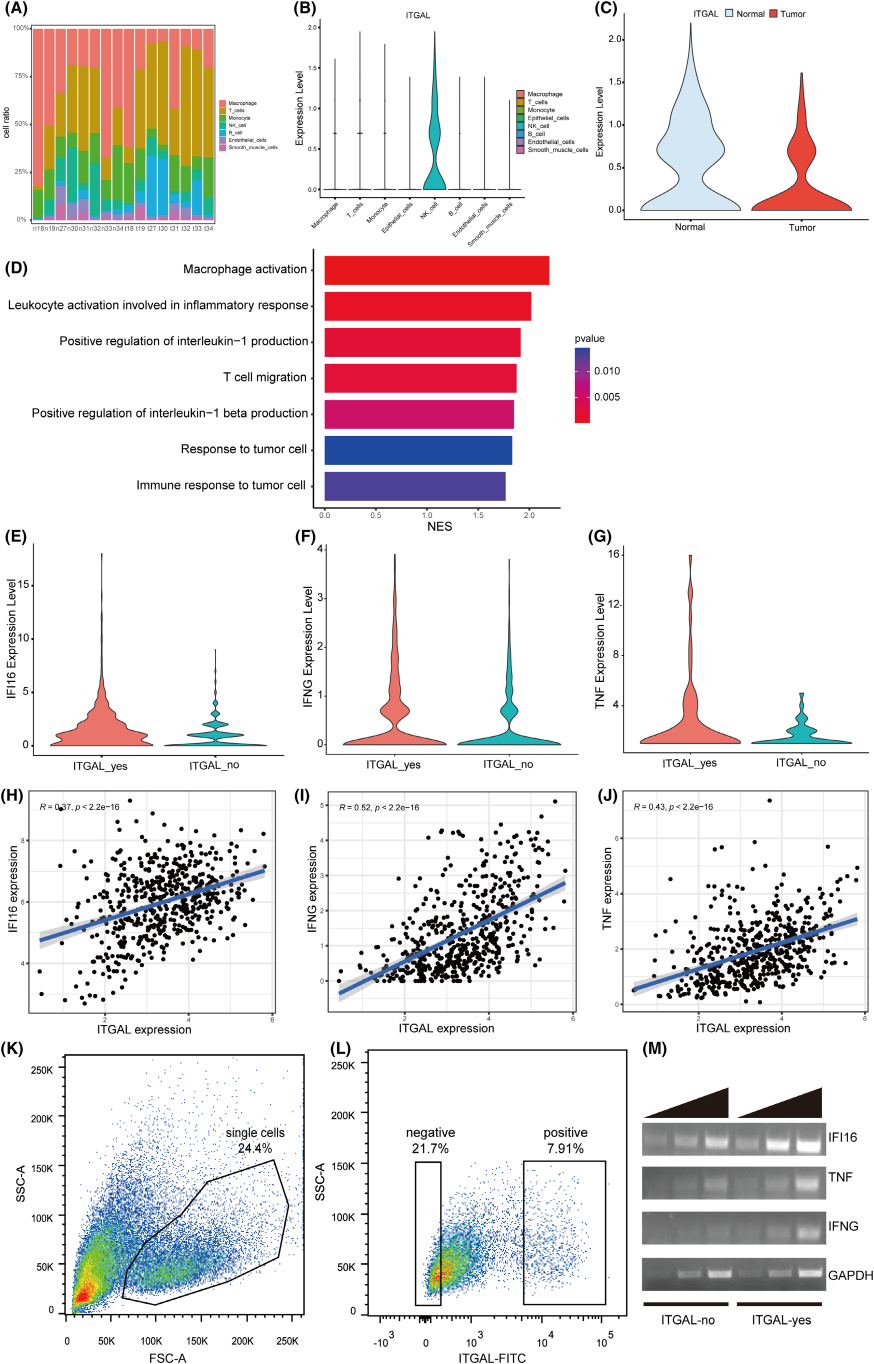
Characterization of ITGAL_yes NK cells in the LUAD. (A) The proportion of each cell type in eight samples. (B) Violin plots showing the expression of the ITGAL in different cell types. (C) Violin plots show the differences of ITGAL expression in NK cells between normal and tumour samples. (D) GSEA analysis showing the enriched pathways correlating positively with the expression of ITGAL in NK cells of tumour samples. (E–G) Violin plots show the differences in IFI16 (E), IFNG (F) and TNF(G) expression between ITGAL_yes and ITGAL_no cells. (H–J) Gene expression correlation between ITGAL and IFI16 (H), IFNG (I) as well as TNF (J) in the LUAD patients from the TCGA dataset. (K, L) Gating strategy for flow cytometric sorting of NK92 cells with high and low ITGAL expression. (M) RT‐PCR of IFI16, IFNG, TNF and GAPDH.

Based on the analysis of scRNA‐seq, we compared the relative abundance of different cell types in the LUAD microenvironment, and found that most LUAD samples had fewer total NK cells than the paired normal samples. We also found that ITGAL was highly expressed in NK cells (Figure 7A,B; Figure S2C). Moreover, subcluster analysis of the NK cells revealed that the expression levels of ITGAL was higher in normal tissues than in tumours (Figure 7C; Figure S2D,E). These results suggested a central role of NK cells in ITGAL‐mediated immune signalling.

To confirm the involvement of ITGAL in NK cells of the LUAD, we sought to identify the ITGAL‐expressing cell subtypes among NK cells. We ranked the differentially expressed genes in ITGAL‐expressing NK cells (ITGAL_yes) compared with the other NK cells (ITGAL_no) by the log2‐transformed fold change values and applied GSEA to identify the differential functional gene sets. The enriched upregulated signalling pathways in ITGAL_yes NK subpopulation were related to positive regulation of interleukin‐1 production, leukocyte activation involved in inflammatory response and immune response to tumour cell (Figure 7D). Specifically, differentially expressed gene analysis identified the cytokines IFI16, IFNG and TNF as significantly upregulated genes in the ITGAL_yes NK subpopulation compared to the ITGAL_no NK subpopulation (Figure 7E–G). Analysis of the TCGA‐LUAD dataset also revealed the positive correlation between ITGAL and IFI16, IFNG, as well as TNF in LUAD (Figure 7H–J). To further confirm the function of NK cell, we examined the expression of ITGAL in NK92 cell line. FACS analysis showed heterogeneous ITGAL expression in NK92 cells (Figure 7K,L). We next purified ITGAL_yes and ITGAL_no NK92 cells, and RT‐PCR confirmed the higher level of IFI16, IFNG and TNF expression in ITGAL_yes NK92 subpopulation (Figure 7M). Consistent with these results, previous studies have found that IFI16, IFNG and TNF play a significant role in immune response, with expression being closely related to the occurrence and development of various tumors.^23^, ^24^, ^25^ Above all, these results suggested that ITGAL expression might affect the TME through regulating the expression of cytokines in NK cells in LUAD.

### 3.8 Prediction of the immunotherapy drugs for LUAD based on the ITGAL expression

**FIGURE 8:**
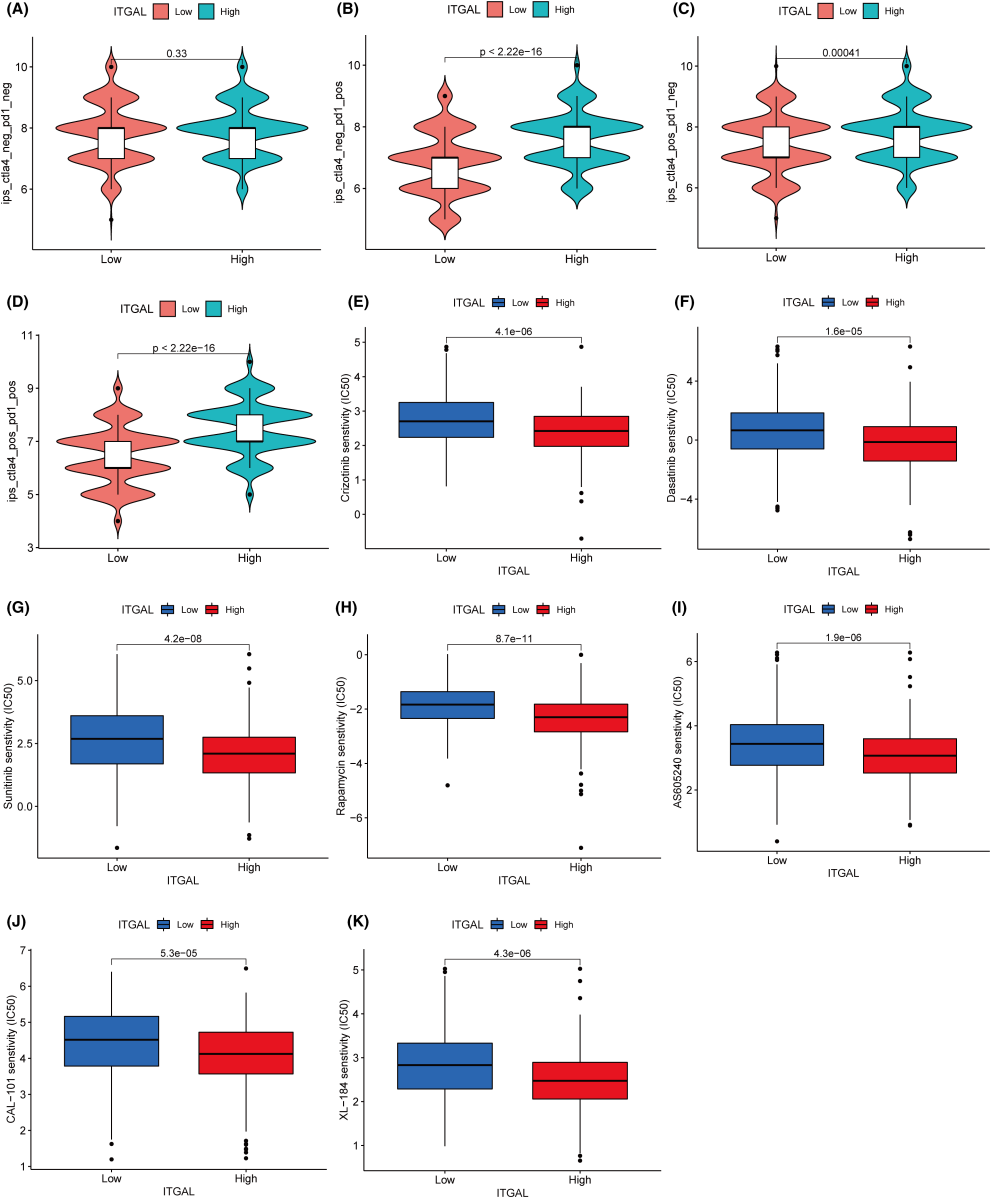
Identification of candidate drugs for LUAD. (A–D) The correlation of ITGAL with CTLA4 and PD1. (E–K) Sensitivity analysis of key drugs in high‐ and low‐expression groups.

The immunophenoscore (IPS) was used to predict the sensitivity of ITGAL expression to immunotherapy drugs such as PD‐1 and CTLA4. This analysis showed that among patients with PD‐1 or CTLA4 positivity, patients with high expression of ITGAL had higher IPS scores than those with low expression, indicating that the expression of ITGAL may promote the effect of immunotherapy (Figure 8A–D). Moreover, to identify more specific drug candidates for LUAD, we used pRRophetic package to predict the potential drug responses in LUAD. The drug sensitivity analysis showed higher sensitivity in the ITGAL high‐expression group than in the low‐expression group, regardless of whether chemotherapy, targeted or immunotherapy drugs were used, indicating that patients with ITGAL high expression of LUAD may benefit significantly from drug therapy (Figure 8E–J).

### 3.9 Validation of expression and prognosis in LUAD samples

**FIGURE 9:**
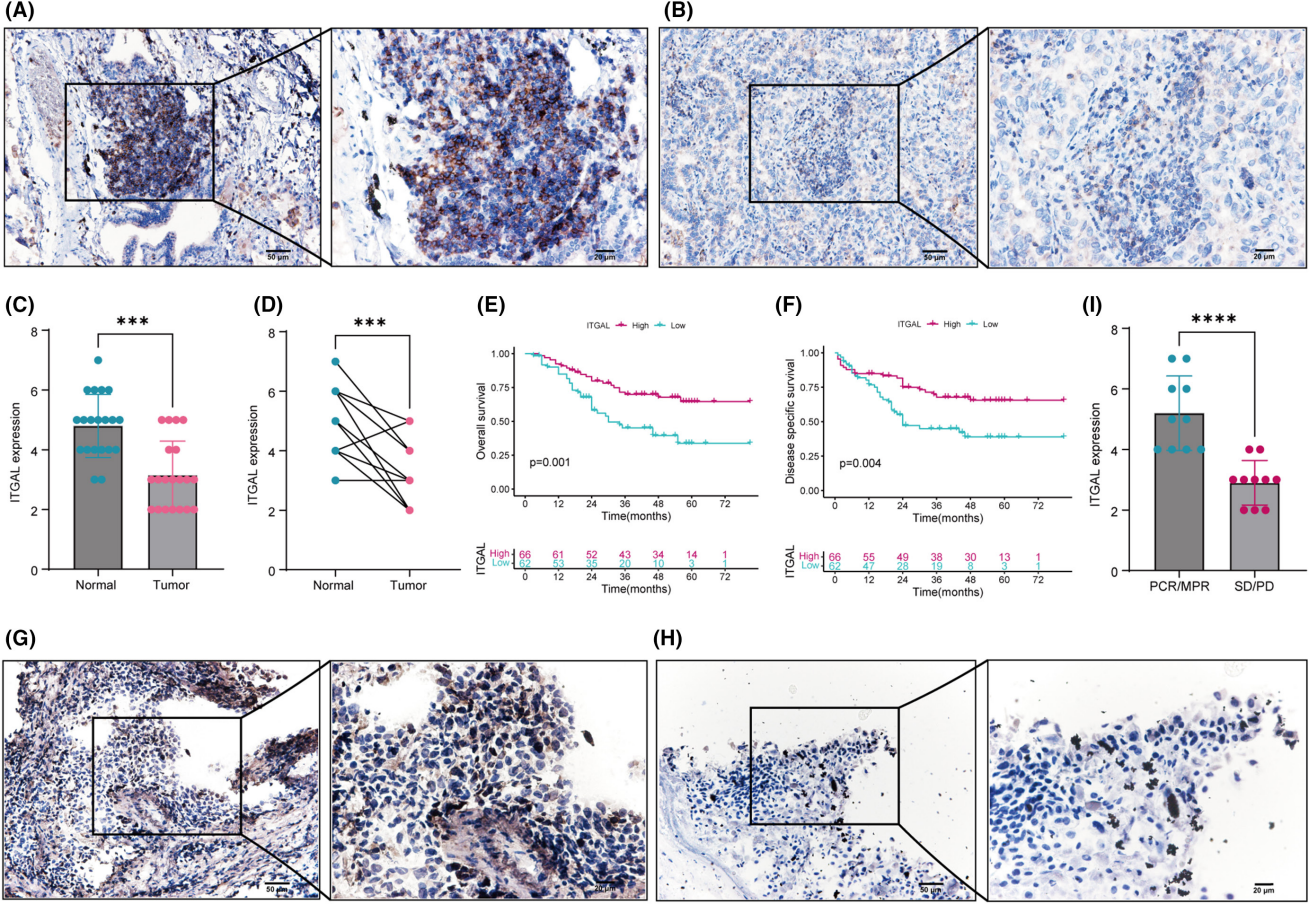
Validation of ITGAL in clinical samples. (A, B) Immunohistochemistry (IHC) staining with anti‐ITGAL antibody was performed on normal lung tissues adjacent to tumours (A) and LUAD tissue (B). Scale bars represent 20 μm. (C) Boxplot shows the expression of ITGAL based on (A, B) ***p < 0.001.D. ITGAL expression between paired tumour and normal samples from the scRNA‐seq cohort in Figure 6, ***p < 0.001. (E, F) Kaplan–Meier plots of overall survival (E) and disease specific survival (F) in LUAD patients based on the expression levels of ITGAL. The frequency of samples with no (0), low (0.1–3.9), or high (4.0–8.0) ITGAL staining stratified by IHC‐defined lung cancer subtype. (G, H) Immunohistochemistry (IHC) staining with anti‐ITGAL antibody was performed on MPR (G) and PD (H). Scale bars represent 20 μm. (I) Boxplot shows the expression of ITGAL based on (G, H) ****p < 0.001.

To validate the prognostic significance of LUAD, we examined the expression levels in resected NSCLC tumours from human subjects with known clinical outcomes. We collected 20 patients from our institution for immunohistochemical verification of ITGAL expression in normal and tumour tissues, and the results showed that the expression of ITGAL was significantly higher in normal tissues (Figure 9A–D). At the same time, we collected tumour tissue from 128 patients for immunohistochemistry, and the results showed that patients with high expression of ITGAL had better OS and DSS (Figure 9E,F). These results confirmed the pivotal role of ITAGL in LUAD, and were consistent with the above findings.

### 3.10 Validation of immunotherapy in LUAD patients

Furthermore, we collected postoperative specimens from 20 patients receiving PD‐1/PD‐L1 treatment, including 10 patients with pathologic complete response (PCR)/major pathologic response (MPR) and 10 patients with stable disease (SD)/progressive disease (PD). Immunohistochemical results showed that ITGAL expression in the PCR/MPR group was significantly higher than that in the SD/PD group (Figure 9G–I), indicating that ITGAL expression plays an important role in immunotherapy, consistent with the above findings.

## 4 DISCUSSION

Numerous studies have demonstrated the potential of ITGAL as a molecular marker for immunotherapy in various tumors,^26^ However, its role in lung adenocarcinoma has seldom been explored. In this study, we observed high expression of ITGAL in normal LUAD tissues using the TCGA dataset, and we found that patients with elevated ITGAL expression exhibited better prognosis. Moreover, we clearly show that ITGAL expression represses the progression of LUAD cancer cells. Functional enrichment analysis revealed a correlation between ITGAL and immune cell infiltration, while single‐cell data indicated predominant expression of ITGAL in NK cells, which was significantly associated with tumour immune invasion and response to immunotherapy. Notably, immunohistochemical analysis confirmed high ITGAL expression in normal tissues, and patients with elevated expression showed improved prognosis. Furthermore, among patients undergoing immunotherapy, those with a favourable treatment response (PCR/MPR) exhibited higher ITGAL infiltration in tumour tissues compared to those with the SD/PD. These findings suggest that ITGAL may represent a novel target for LUAD therapy.

TME plays an important role in the occurrence and development of LUAD.^27^, ^28^ The nature of the interaction of tumour cells with immune cells in the TME defines the antitumor response.^29^ Immune checkpoints, which are proteins produced by some immune cells (e.g. T cells) and cancer cells, is a vital factor influencing immunotherapy efficacy. Recent studies have discovered several immune checkpoints in LUAD, including programmed death‐1 (PD‐1), T‐cell immunoglobulin domain and mucin domain‐containing molecule‐3 (TIM‐3), T‐cell immunoglobulin and ITIM domain (TIGIT), B and T cell lymphocyte attenuator (BTLA), lymphocyte activation gene (LAG3), V‐domain Ig suppressor of T cell activation (VISTA) and Cluster of Differentiation 200 (CD200),^30^ however, immunotherapy efficacy is limited by the lack of reliable biomarkers to identify potential therapeutic‐responsive patients in LUAD,^29^, ^31^, ^32^ and as a result, a substantial number of patients do not benefit from immunotherapy.^33^, ^34^ Cancer immunotherapy would elicit a cellular immune response, especially the T‐cell‐mediated immune response, which can selectively destroy a tumor.^35^ In addition to T cells, NK cells are among the principal mediators of the immunological response against malignant cells,^36^, ^37^ however, the molecular mechanism of NK cells in LUAD remains relatively poorly understood. ITGAL, a tissue‐specific integrin, contributes to inflammatory and immune responses.^38^ Previous investigations have highlighted the ability of ITGAL to enhance T‐cell migration through direct contact with CLL cells, involving the suppression of Rho GTPase signaling.^39^, ^40^ Our findings demonstrated a significant correlation between ITGAL expression and NK cells in LUAD, suggesting their involvement in immune response‐related cell activation within tumours.

We conducted a comprehensive analysis of bulk RNA‐seq and scRNA‐seq to investigate the role of ITGAL in LUAD. For one thing, high expression of ITGAL inhibits the malignant progression of tumour cells; for another, ITGAL plays a crucial role in recruiting and modulating NK cells in LUAD, which could promote the secretion of cytokines in NK cells. NK cells with high expression of ITGAL produce more cytokines, which may also be the reason for their sensitivity to immunotherapy drugs. In addition, the expression of ITGAL in different patients (tumour vs. normal; PCR/MPR vs. SD/PD) can be effectively distinguished through immunohistochemistry. Our study provides new theoretical insights into the role of ITGAL in the prognosis and precision therapy of LUAD patients.

This study reveals that ITGAL is associated with outcome of LUAD, ITGAL not only exerts an impact on the behaviour of LUAD cells but also induces modifications in the immune microenvironment. However, this study has several limitations. First, the majority of the data utilized in this research are sourced from online platform databases, which undergo continuous updates and expansions. Consequently, the findings may be subject to potential impact. Second, the functional role of ITGAL in LUAD and its molecular mechanism within the tumour microenvironment have not been validated through in vivo experiments. Finally, drug sensitivity needs further confirmation by cell experiment. It is necessary to carry out large‐sample trials and incorporate experiments for verification in the future.

## 5 CONCLUSIONS

ITGAL is a prognostic biomarker for LUAD patients, and it exhibits a dual function in LUAD by not only suppressing the malignant progression of tumour cells but also modulating immunotherapy through NK cells. Therefore, ITGAL may be an important target in LUAD therapy.

## AUTHOR CONTRIBUTIONS

**Zengtuan Xiao:** Data curation (equal); formal analysis (equal); investigation (equal); resources (equal); software (equal). **Zhe Nian:** Formal analysis (equal); investigation (equal); validation (equal); writing – original draft (equal). **Mengzhe Zhang:** Data curation (equal); investigation (equal); validation (equal). **Zuo Liu:** Data curation (equal); supervision (equal); validation (equal). **Zhe Liu:** Project administration (equal); writing – review and editing (equal). **Zhenfa Zhang:** Funding acquisition (lead); project administration (lead); writing – review and editing (lead).

## FUNDING INFORMATION

This study was supported by the National Natural Science Foundations of China (Number: 82273119).

## CONFLICT OF INTEREST STATEMENT

The authors declare no conflicts of interest.

## Supporting information


**Figure S1.** Association of ITGAL with clinical features and immunotherapy targets. (A) ITGAL mRNA level in paired tumour samples in the LUAD based on TCGA dataset, ***p < 0.001. (B) ITGAL mRNA level in paired tumour samples in the LUAD based on GEO databases (GSE140343), ****p < 0.0001. (C) Boxplots depict the expression of ITGAL in patients of the TCGA dataset, as categorized based on gender. (D) The correlation between ITGAL and N stage in TCGA dataset; (E) The correlation between ITGAL and M stage in TCGA dataset; (F) Heat map of ITGAL correlation with clinical features; (G) Kaplan–Meier plots of disease‐free survival; (H, I). Heat map of ITGAL correlation with immunotherapy targets.

**Figure S2.** Single cell RNA‐seq of LUAD. (A) Histograms showing the distribution of total nFeature, nCount, mitochondrial reads per cell and haemoglobin reads per cell. (B) Uniform manifold approximation and projection (UMAP) plots for the 99,867 high‐quality cells showing sample type. (C) The proportion of each cell type in normal and tumour samples. (D, E) The average expression of ITGAL based on the single cell datasets. **p < 0.01, ****p < 0.0001.

Table S1.

## Data Availability

The data that support the findings of this study are available in TCGA, GEO dataset and 10.24433/CO.0121060.v1 (reference:20). The original contributions presented in the study are included in the article/Supplementary Materials, further inquiries can be directed to the corresponding authors.

## References

[1] Siegel RL , Miller KD , Wagle NS , Jemal A . Cancer statistics, 2023. CA Cancer J Clin. 2023;73(1):17‐48. 36633525 10.3322/caac.21763

[2] Duma N , Santana‐Davila R , Molina JR . Non‐small cell lung cancer: epidemiology, screening, diagnosis, and treatment. Mayo Clin Proc. 2019;94(8):1623‐1640. 31378236 10.1016/j.mayocp.2019.01.013

[3] Lin JJ , Cardarella S , Lydon CA , et al. Five‐year survival in EGFR‐mutant metastatic lung adenocarcinoma treated with EGFR‐TKIs. J Thorac Oncol. 2016;11(4):556‐565. 26724471 10.1016/j.jtho.2015.12.103 PMC4979601

[4] Sharma P , Allison JP . The future of immune checkpoint therapy. Science. 2015;348(6230):56‐61. 25838373 10.1126/science.aaa8172

[5] Sharma P , Siddiqui BA , Anandhan S , et al. The next decade of immune checkpoint therapy. Cancer Discov. 2021;11(4):838‐857. 33811120 10.1158/2159-8290.CD-20-1680

[6] Gibney GT , Weiner LM , Atkins MB . Predictive biomarkers for checkpoint inhibitor‐based immunotherapy. Lancet Oncol. 2016;17(12):e542‐e551. 27924752 10.1016/S1470-2045(16)30406-5 PMC5702534

[7] Anagnostou V , Smith KN , Forde PM , et al. Evolution of neoantigen landscape during immune checkpoint blockade in non‐small cell lung cancer. Cancer Discov. 2017;7(3):264‐276. 28031159 10.1158/2159-8290.CD-16-0828 PMC5733805

[8] Chan TA , Wolchok JD , Snyder A . Genetic basis for clinical response to CTLA‐4 blockade in melanoma. N Engl J Med. 2015;373(20):1984. 26559592 10.1056/NEJMc1508163

[9] Goodman AM , Kato S , Bazhenova L , et al. Tumor mutational burden as an independent predictor of response to immunotherapy in diverse cancers. Mol Cancer Ther. 2017;16(11):2598‐2608. 28835386 10.1158/1535-7163.MCT-17-0386 PMC5670009

[10] Sharma P , Hu‐Lieskovan S , Wargo JA , Ribas A . Primary, adaptive, and acquired resistance to cancer immunotherapy. Cell. 2017;168(4):707‐723. 28187290 10.1016/j.cell.2017.01.017 PMC5391692

[11] Takada Y , Ye X , Simon S . The integrins. Genome Biol. 2007;8(5):215. 17543136 10.1186/gb-2007-8-5-215 PMC1929136

[12] Desgrosellier JS , Cheresh DA . Integrins in cancer: biological implications and therapeutic opportunities. Nat Rev Cancer. 2010;10(1):9‐22. 20029421 10.1038/nrc2748 PMC4383089

[13] Seguin L , Desgrosellier JS , Weis SM , Cheresh DA . Integrins and cancer: regulators of cancer stemness, metastasis, and drug resistance. Trends Cell Biol. 2015;25(4):234‐240. 25572304 10.1016/j.tcb.2014.12.006 PMC4380531

[14] Cooper J , Giancotti FG . Integrin signaling in cancer: mechanotransduction, stemness, epithelial plasticity, and therapeutic resistance. Cancer Cell. 2019;35(3):347‐367. 30889378 10.1016/j.ccell.2019.01.007 PMC6684107

[15] Zhang J , Wang H , Yuan C , et al. ITGAL as a prognostic biomarker correlated with immune infiltrates in gastric cancer. Front Cell Dev Biol. 2022;10:808212. 35399517 10.3389/fcell.2022.808212 PMC8987306

[16] De Andrade CA , Chatterjee J , Cobb O , et al. RNA sequence analysis reveals ITGAL/CD11A as a stromal regulator of murine low‐grade glioma growth. Neuro‐Oncology. 2022;24(1):14‐26. 34043012 10.1093/neuonc/noab130 PMC8730775

[17] Rojas K , Baliu‐Piqué M , Manzano A , et al. In silico transcriptomic mapping of integrins and immune activation in basal‐like and HER2+ breast cancer. Cell Oncol (Dordr). 2021;44(3):569‐580. 33469836 10.1007/s13402-020-00583-9 PMC12980691

[18] Vendrell E , Ribas M , Valls J , et al. Genomic and transcriptomic prognostic factors in R0 dukes B and C colorectal cancer patients. Int J Oncol. 2007;30(5):1099‐1107. 17390011

[19] Song Y , Pan Y , Liu J . The relevance between the immune response‐related gene module and clinical traits in head and neck squamous cell carcinoma. Cancer Manag Res. 2019;11:7455‐7472. 31496804 10.2147/CMAR.S201177 PMC6689548

[20] Bischoff P , Trinks A , Obermayer B , et al. Single‐cell RNA sequencing reveals distinct tumor microenvironmental patterns in lung adenocarcinoma. Oncogene. 2021;40(50):6748‐6758. 34663877 10.1038/s41388-021-02054-3 PMC8677623

[21] Mayakonda A , Lin DC , Assenov Y , Plass C , Koeffler HP . Maftools: efficient and comprehensive analysis of somatic variants in cancer. Genome Res. 2018;28(11):1747‐1756. 30341162 10.1101/gr.239244.118 PMC6211645

[22] Charoentong P , Finotello F , Angelova M , et al. Pan‐cancer immunogenomic analyses reveal genotype‐immunophenotype relationships and predictors of response to checkpoint blockade. Cell Rep. 2017;18(1):248‐262. 28052254 10.1016/j.celrep.2016.12.019

[23] Ouchi M , Ouchi T . Role of IFI16 in DNA damage and checkpoint. Front Biosci. 2008;13:236‐239. 17981541 10.2741/2673

[24] Rotman J , den Otter LAS , Bleeker MCG , et al. PD‐L1 and PD‐L2 expression in cervical cancer: regulation and biomarker potential. Front Immunol. 2020;11:596825. 33424844 10.3389/fimmu.2020.596825 PMC7793653

[25] Jiang Y , Chen J , Bi E , et al. TNF‐α enhances Th9 cell differentiation and antitumor immunity via TNFR2‐dependent pathways. J Immunother Cancer. 2019;7(1):28. 30717817 10.1186/s40425-018-0494-8 PMC6360681

[26] Li J , Du Q , Sun J , et al. Identification and validation of a novel phagocytosis regulators‐related signature with potential prognostic and immunotherapeutic value in patients with lung adenocarcinoma. Front Oncol. 2022;12:988332. 36408131 10.3389/fonc.2022.988332 PMC9666737

[27] Bhargava A , Mishra DK , Tiwari R , Lohiya NK , Goryacheva IY , Mishra PK . Immune cell engineering: opportunities in lung cancer therapeutics. Drug Deliv Transl Res. 2020;10(5):1203‐1227. 32172351 10.1007/s13346-020-00719-2

[28] Genova C , Dellepiane C , Carrega P , et al. Therapeutic implications of tumor microenvironment in lung cancer: focus on immune checkpoint blockade. Front Immunol. 2021;12:799455. 35069581 10.3389/fimmu.2021.799455 PMC8777268

[29] Mi K , Chen F , Qian Z , et al. Characterizing heterogeneity of non‐small cell lung tumour microenvironment to identify signature prognostic genes. J Cell Mol Med. 2020;24(24):14608‐14618. 33184998 10.1111/jcmm.16092 PMC7754023

[30] Salehi‐Rad R , Li R , Paul MK , Dubinett SM , Liu B . The biology of lung cancer: development of more effective methods for prevention, diagnosis, and treatment. Clin Chest Med. 2020;41(1):25‐38. 32008627 10.1016/j.ccm.2019.10.003

[31] Lahiri A , Maji A , Potdar PD , et al. Lung cancer immunotherapy: progress, pitfalls, and promises. Mol Cancer. 2023;22(1):40. 36810079 10.1186/s12943-023-01740-y PMC9942077

[32] Raez LE , Cassileth PA , Schlesselman JJ , et al. Allogeneic vaccination with a B7.1 HLA‐A gene‐modified adenocarcinoma cell line in patients with advanced non‐small‐cell lung cancer. J Clin Oncol. 2004;22(14):2800‐2807. 15254047 10.1200/JCO.2004.10.197

[33] Camidge DR , Doebele RC , Kerr KM . Comparing and contrasting predictive biomarkers for immunotherapy and targeted therapy of NSCLC. Nat Rev Clin Oncol. 2019;16(6):341‐355. 30718843 10.1038/s41571-019-0173-9

[34] Suresh K , Naidoo J , Lin CT , Danoff S . Immune checkpoint immunotherapy for non‐small cell lung cancer: benefits and pulmonary toxicities. Chest. 2018;154(6):1416‐1423. 30189190 10.1016/j.chest.2018.08.1048 PMC6335259

[35] Pardoll D . Does the immune system see tumors as foreign or self? Annu Rev Immunol. 2003;21:807‐839. 12615893 10.1146/annurev.immunol.21.120601.141135

[36] Klingemann H , Boissel L , Toneguzzo F . Natural killer cells for immunotherapy—advantages of the NK‐92 cell line over blood NK cells. Front Immunol. 2016;7:91. 27014270 10.3389/fimmu.2016.00091 PMC4789404

[37] Sakamoto N , Ishikawa T , Kokura S , et al. Phase I clinical trial of autologous NK cell therapy using novel expansion method in patients with advanced digestive cancer. J Transl Med. 2015;13:277. 26303618 10.1186/s12967-015-0632-8 PMC4548900

[38] Lu Q , Ray D , Gutsch D , Richardson B . Effect of DNA methylation and chromatin structure on ITGAL expression. Blood. 2002;99(12):4503‐4508. 12036881 10.1182/blood.v99.12.4503

[39] Ramsay AG , Evans R , Kiaii S , Svensson L , Hogg N , Gribben JG . Chronic lymphocytic leukemia cells induce defective LFA‐1‐directed T‐cell motility by altering rho GTPase signaling that is reversible with lenalidomide. Blood. 2013;121(14):2704‐2714. 23325833 10.1182/blood-2012-08-448332 PMC3617635

[40] Haspels HN , Rahman MA , Joseph JV , Gras Navarro A , Chekenya M . Glioblastoma stem‐like cells are more susceptible than differentiated cells to natural killer cell lysis mediated through killer immunoglobulin‐like receptors‐human leukocyte antigen ligand mismatch and activation receptor‐ligand interactions. Front Immunol. 2018;9:1345. 29967607 10.3389/fimmu.2018.01345 PMC6015895

