# Supplementary figures and images for: Integrated analysis highlights the significance role of ITGAL in lung adenocarcinoma
Source: J Cell Mol Med. 2024 Apr 13;28(8):e18289. doi: 10.1111/jcmm.18289 (PMC11015394; doi:10.1111/jcmm.18289)

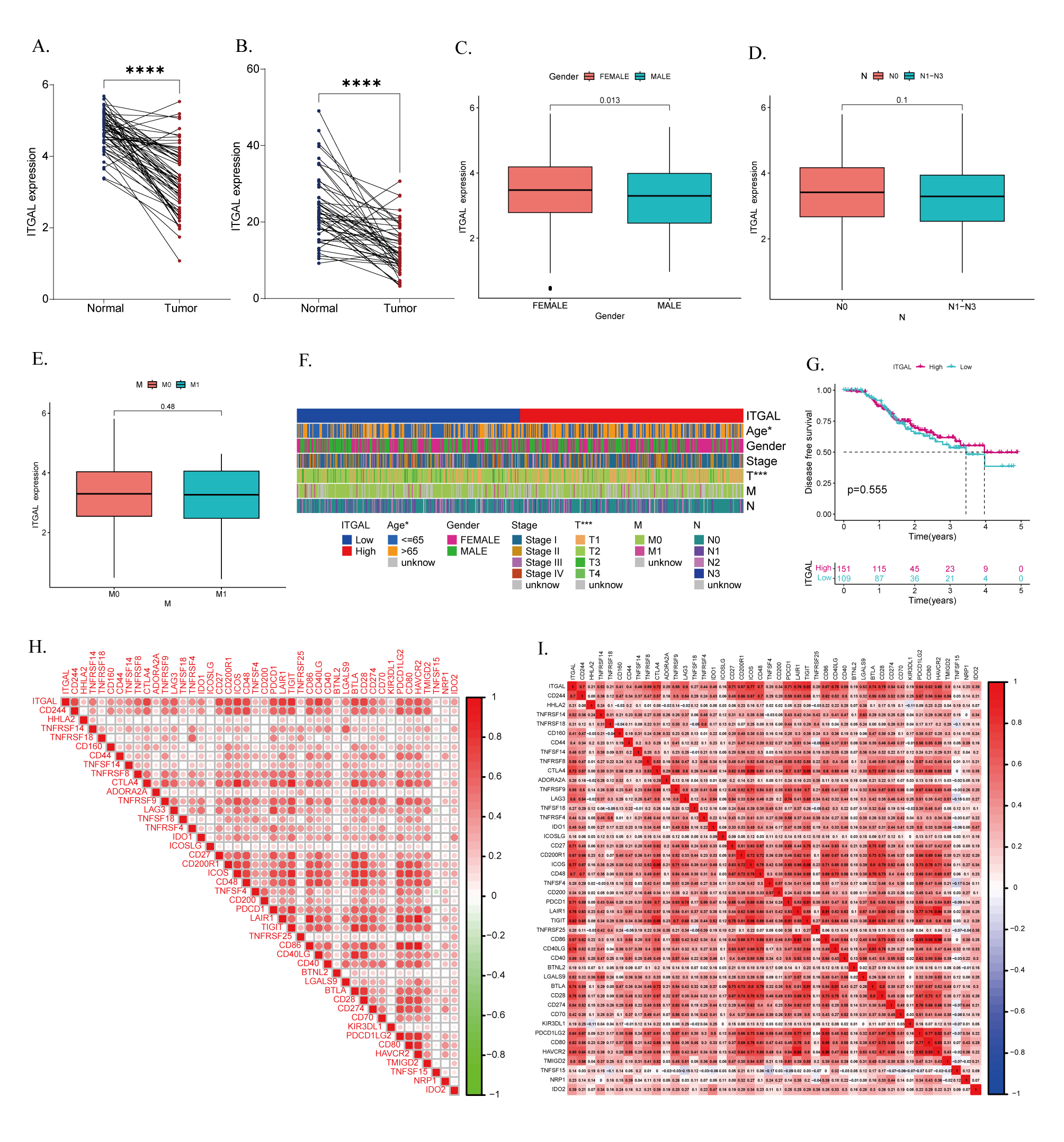

Supplement: Supplementary file 1 — Figure S1. Association of ITGAL with clinical features and immunotherapy targets. (A) ITGAL mRNA level in paired tumour samples in the LUAD based on TCGA dataset, ***p < 0.001. (B) ITGAL mRNA level in paired tumour samples in the LUAD based on GEO databases (GSE140343), ****p < 0.0001. (C) Boxplots depict the expression of ITGAL in patients of the TCGA dataset, as categorized based on gender. (D) The correlation between ITGAL and N stage in TCGA dataset; (E) The correlation between ITGAL and M stage in TCGA dataset; (F) Heat map of ITGAL correlation with clinical features; (G) Kaplan–Meier plots of disease‐free survival; (H, I). Heat map of ITGAL correlation with immunotherapy targets. [file JCMM-28-e18289-s002.tif]

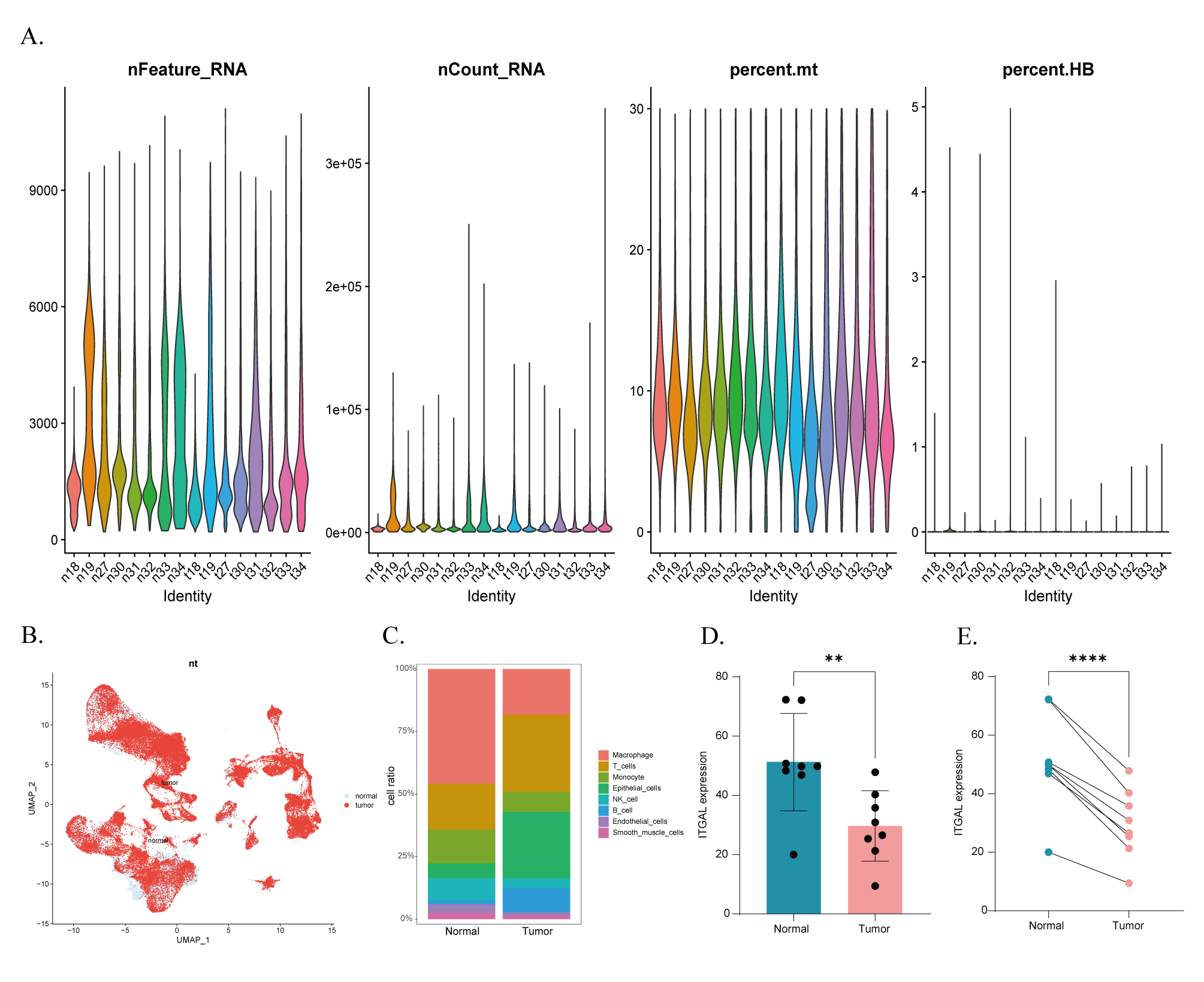

Supplement: Supplementary file 2 — Figure S2. Single cell RNA‐seq of LUAD. (A) Histograms showing the distribution of total nFeature, nCount, mitochondrial reads per cell and haemoglobin reads per cell. (B) Uniform manifold approximation and projection (UMAP) plots for the 99,867 high‐quality cells showing sample type. (C) The proportion of each cell type in normal and tumour samples. (D, E) The average expression of ITGAL based on the single cell datasets. **p < 0.01, ****p < 0.0001. [file JCMM-28-e18289-s001.tif]
